# Single‐Cell Transcriptomics Reveal Human Skin Aging Pathways

**DOI:** 10.1111/jocd.70708

**Published:** 2026-04-07

**Authors:** Kseniya Melyukhina, Anne‐Laure Bulteau, Carine Nizard, Karl Pays, Jesse R. Poganik, Vadim N. Gladyshev, Laure Crabbe

**Affiliations:** ^1^ Division of Genetics, Department of Medicine, Brigham and Women's Hospital, Harvard Medical School Boston Massachusetts USA; ^2^ LVMH Recherche Saint Jean de Braye France

**Keywords:** aging, hallmarks, pathways, sc transcriptomics, skin

To the Editor,

1

Skin is the largest organ of the human body that separates internal organs from the external environment and its constant exposure to a variety of stressors. This essential protective role is challenged during organismal aging, as human skin suffers from both intrinsic and extrinsic aging factors, with visible consequences such as wrinkles, loss of elasticity, laxity, and a rough‐textured appearance. These changes translate to phenotypic deviations in cutaneous cells and on the extracellular matrix (ECM) components that are essential for skin physical properties and hydration. A previous study estimated that over 80% of the elderly experience some types of dermatological condition [1], a finding consistent with the intricate interplay between the hallmarks of aging in the skin and systemic biological aging [2]. Preserving skin health during organismal aging is an essential challenge to ensure skin plays its protective role.

Here, we aimed to characterize the aging signature of human skin dermal fibroblasts (FB) and epidermal keratinocytes (KT) using single‐cell transcriptome sequencing and bioinformatics analysis. Skin samples from five young donors (33 ± 2 years old) and five aged donors (74 ± 2 years old) were collected for this analysis (Table S1) [3]. All participants provided informed consent.

Skin samples were processed using a standardized tissue dissociation method to obtain viable single cell suspensions. More than 80 000 cells were sequenced and annotated. They clustered into thirteen different cell types using the dimension reduction method Uniform Manifold Approximation and Projection (UMAP) (Figure 1A). As expected, FB and KT were the most abundant cell types present in the dataset and revealed a clearly defined transcriptomic signature (Figure 1A,B). FB expressed high levels of Collagen Type I Alpha I Chain (COL1A1) and Alpha 2 Chain (COL1A2), Decorin (DCN), Fibrillin (FBN), and Elastin (ELN), while KT expressed different types of keratins relative to their differentiation status (Figure 1B). Only a small subset of KT expressed the proliferation marker MKI67, highlighting the subgroup of proliferative epidermal basal cells. UMAP performed on FB from aged and young donors highlighted an age‐dependent transcriptional profile. Aged fibroblasts expressed higher levels of the senescence marker CDKN1A and decreased expression of major dermal extracellular matrix components (Figure 1C). Similarly, KT also formed discernible age‐dependent clusters, which affected the expression of keratins (Figure 1C).

**FIGURE 1:**
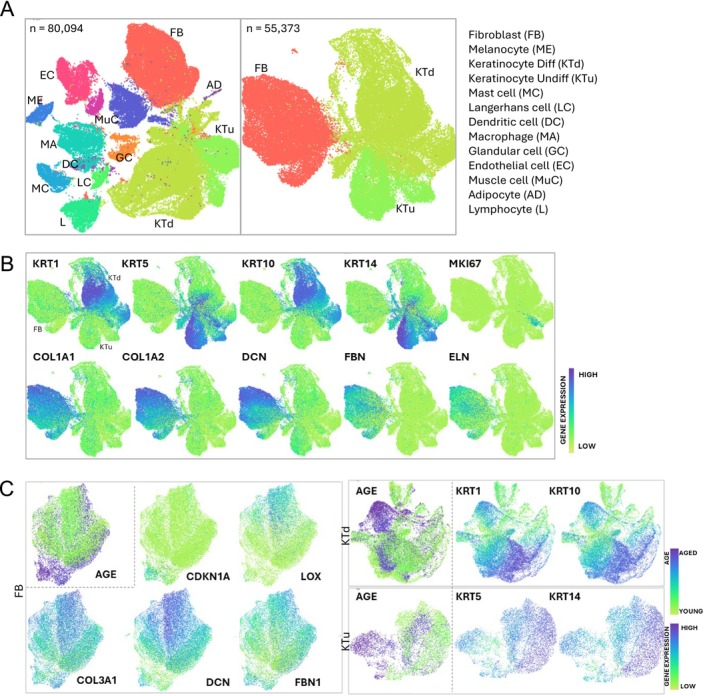
Human skin aging alters the transcriptional profile of skin cells. (A) Uniform manifold approximation and projection (UMAP) plot showing 13 cell types of human skin (left), and a UMAP of only fibroblasts (FB) and keratinocytes (KT). Cells are colored by types and annotated to the right. (B) UMAP plots showing the representative genes of FB and KT in human skin. The color key from green to purple indicates low to high gene expression levels. (C) UMAP plot showing the expression of the indicated genes in FB (left), KTd (top right) and KTu (bottom right). The color key from green to purple indicates low to high gene expression levels, or young to aged cells.

Through these single‐cell transcriptomics dataset, we identified more than 1209 differentially expressed genes (DEGs, |avg_logFC| > 0.58 and p_val_adj < 0.05) in FB, 933 DEGs in differentiated KT, and 855 DEGs in undifferentiated KT when comparing aged samples with young ones. The Venn diagram revealed 384 common DEGs among the different cell types, with a strong similarity between differentiated and undifferentiated KT (Figure S1). Pathway enrichment analysis revealed that age‐dependent DEGs were associated with important aging pathways (Figure S1 and Figure 2A,B). Both FB and KT showed a strong up‐regulation of genes involved in inflammation, and a down‐regulation of genes involved in oxidative stress detoxification (Figure 2C). They also displayed DEGs in the selenium micronutrient pathway, connected to defects in oxidative stress response (Figure 2B–D). Aged skin cells engaged in endoplasmic reticulum stress response, activated the MAP Kinase stress signaling, DNA damage response, and showed impaired mitochondrial oxidative phosphorylation system. An in‐depth analysis of the DEGs also revealed an imbalanced expression of circadian genes (Figure 2E), and an activation of autophagy with an upregulation of mTOR in FB (Figure 2F).

**FIGURE 2:**
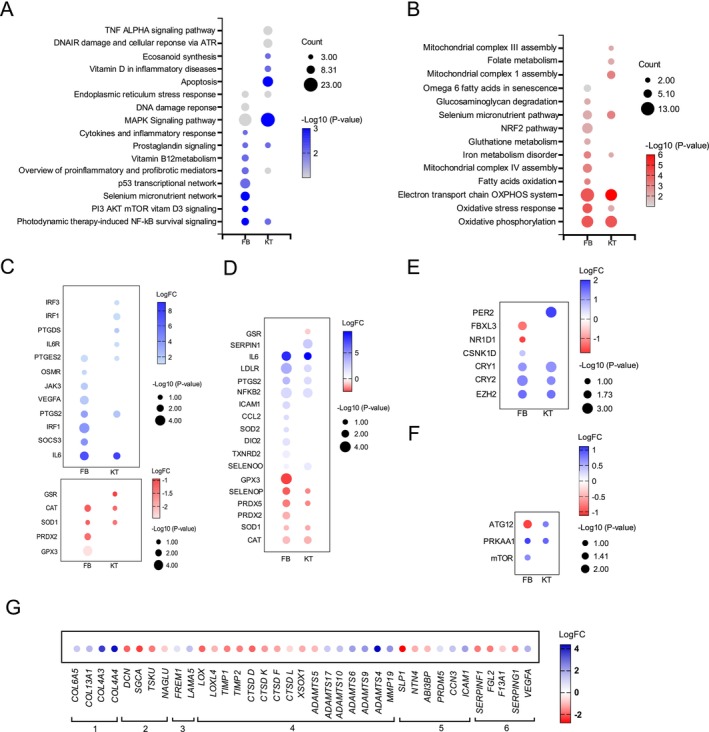
Enriched pathways of aged‐related DEGs in skin cells. (A) Upregulated Wikipathways in FB and KT. “Count” indicates gene number modulated in each pathway. (B) Downregulated Wikipathways in FB and KT. “Count” indicates gene number modulated in each pathway. (C) Age‐dependent upregulated inflammatory genes in FB and KT (top) and age‐dependent downregulated antioxidant genes in FB and KT (bottom). (D) DEGs in FB and KT from the Wikipathway “Selenium micronutrient network”. (E) DEGs in FB and KT related to circadian rhythms. (F) DEGs in FB and KT related to autophagy. (G) DEGs in FB related to skin ECM components: (1) core matrisomal proteins, (2) proteoglycans and glycoproteins, (3) Basement membrane proteins, (4) ECM architecture and remodeling, (5) Matrix associated factors, (6) angiogenesis.

One of the key functions of dermal FB is the production of ECM that provides the proper environment of the cells but also ensures skin mechanical properties. Recent transcriptomics and proteomics studies have enabled the identification of a comprehensive catalog of proteins within the human skin ECM [4, 5, 6]. Analysis of gene regulation responsible for the expression of these factors revealed a significant impact of aging, suggesting a drastic age‐dependent change in ECM composition and maintenance (Figure 2G).

In conclusion, our study provides a transcriptomic aging signature of human skin FB and KT at a single‐cell resolution. Most of the pathways we uncovered were common with the hallmarks of aging described by López‐Otín et al. in Cell in 2023 [7]. This suggests that human skin follows a similar aging path as other organs, with strong hallmarks such as cellular senescence or inflammation. But our study also brought to light new features of skin aging. We uncovered a marked decrease in cellular response to oxidative stress, and changes in the expression of genes regulating the circadian clock. Both cell types shared common aging pathways but also marked specificities. Skin FB extensively changed their expression of ECM genes, which we believe is an important new skin aging hallmark that was also recently added to the list of organismal aging markers [8]. A marker of cellular senescence was observed in FB but not in KT, which activated apoptosis instead. KT also strongly activated the MAP Kinase stress response pathway, much more than what could be noted in FB.

Our study provides a thorough analysis of the consequences of aging using single skin cell transcriptomics. Several limitations should however be noted. First, our methodology does not allow the characterization of three other essential and well‐known hallmarks of skin aging: (i) changes in the epigenetic status of chromatin, (ii) dysbiosis, and (iii) stem cells exhaustion [2]. While all three indirectly affect the transcriptomic profile of FB and KT, these fell outside the scope of our approach. Second, anatomical site and sex distributions differed between our young and aged cohorts. Young donors included samples from abdomen and groin (2 male, 3 female), while aged donors were exclusively female with samples from abdomen and face. Human skin exhibits marked transcriptomic heterogeneity across anatomical sites that reflects physiological and developmental variation [9], and sex‐specific differences in skin aging have been reported. Therefore, some of the age‐associated transcriptomic changes we observed may reflect these confounding variables rather than intrinsic aging effects alone. Future studies with anatomically and sex‐matched cohorts will be essential to definitively establish skin aging signatures.

## Author Contributions

J.R.P.: data curation, methodology, manuscript revision, K.M.: sample collection, A.‐L.B., C.N., K.P.: manuscript revision, V.N.G.: supervision, methodology, L.C.: data analysis, original draft preparation.

## Ethics Statement

This study was approved by the Mass General Brigham Institutional Review Board (protocol #2021P001874).

## Conflicts of Interest

Declaration of competing interest A.‐L.B., C.N., K.P. and L.C. are LVMH recherche employees. The research was funded by LVMH research to perform research at Brigham and Women's Hospital. V.N.G. is an Advisor on the Reverse Aging Science Board at Parfums Christian Dior. Other authors declare no conflicts of interest.

## Supporting information


**FIGURE S1:** (A) Venn view of DEGs from FB (blue), KTd (yellow) and Ktu (purple). (B) Enriched Wikipathways in FB (HNF) and KT (HNK). “Count” indicate gene number modulated in each pathway.
**TABLE S1:** Description of the skin samples enrolled in this study.

## Data Availability

The data that support the findings of this study are available from the corresponding author upon reasonable request.
