# Supplementary material for: Single‐Cell Transcriptomics Reveal Human Skin Aging Pathways
Source: J Cosmet Dermatol. 2026 Apr 7;25(Suppl 1):e70708. doi: 10.1111/jocd.70708 (PMC13055572; doi:10.1111/jocd.70708)

## Slide 1
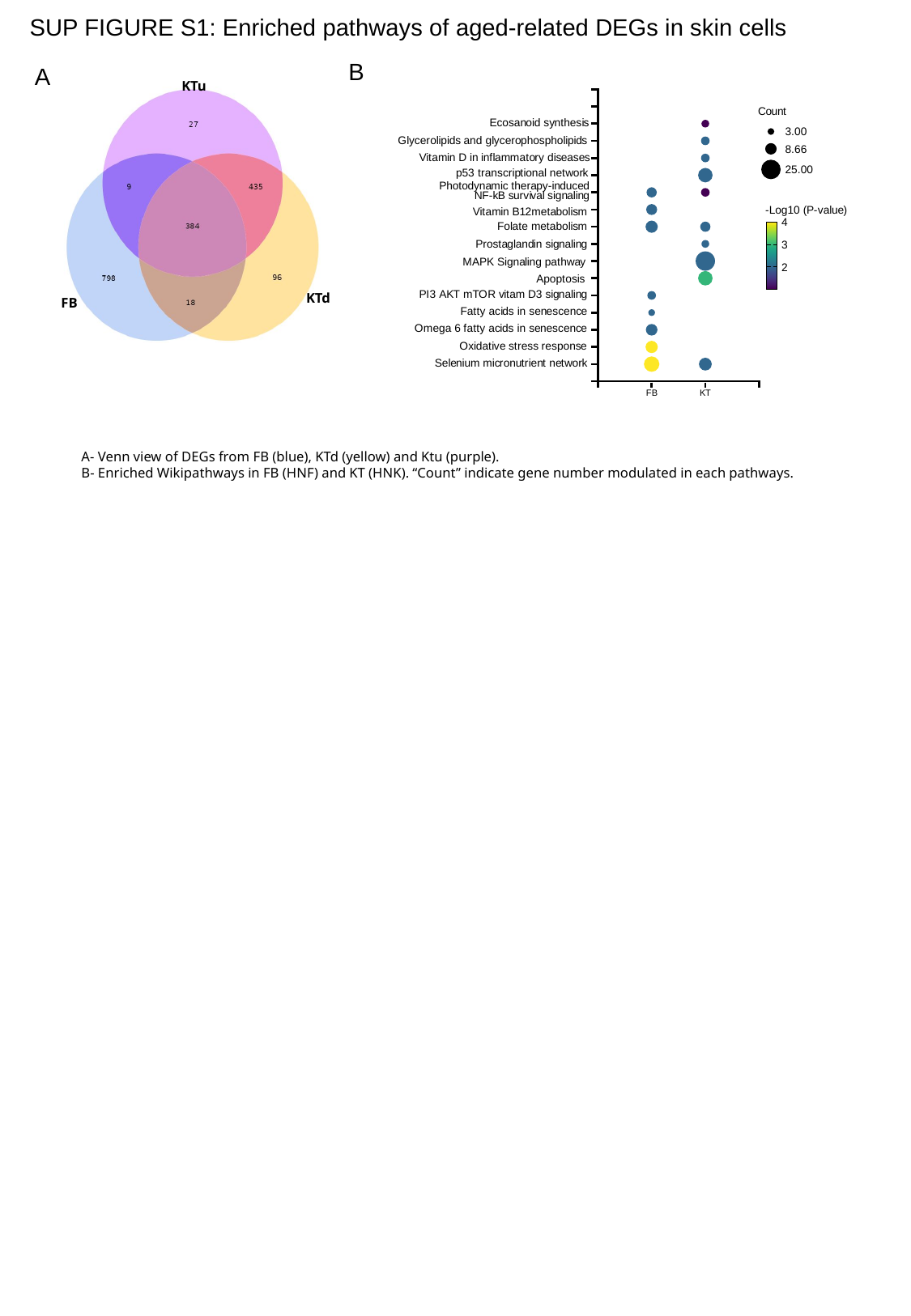

SUP FIGURE S1: Enriched pathways of aged-related DEGs in skin cells
B
A
KTu
KTd
FB
A- Venn view of DEGs from FB (blue), KTd (yellow) and Ktu (purple).
B- Enriched Wikipathways in FB (HNF) and KT (HNK). “Count” indicate gene number modulated in each pathways.

## Slide 2
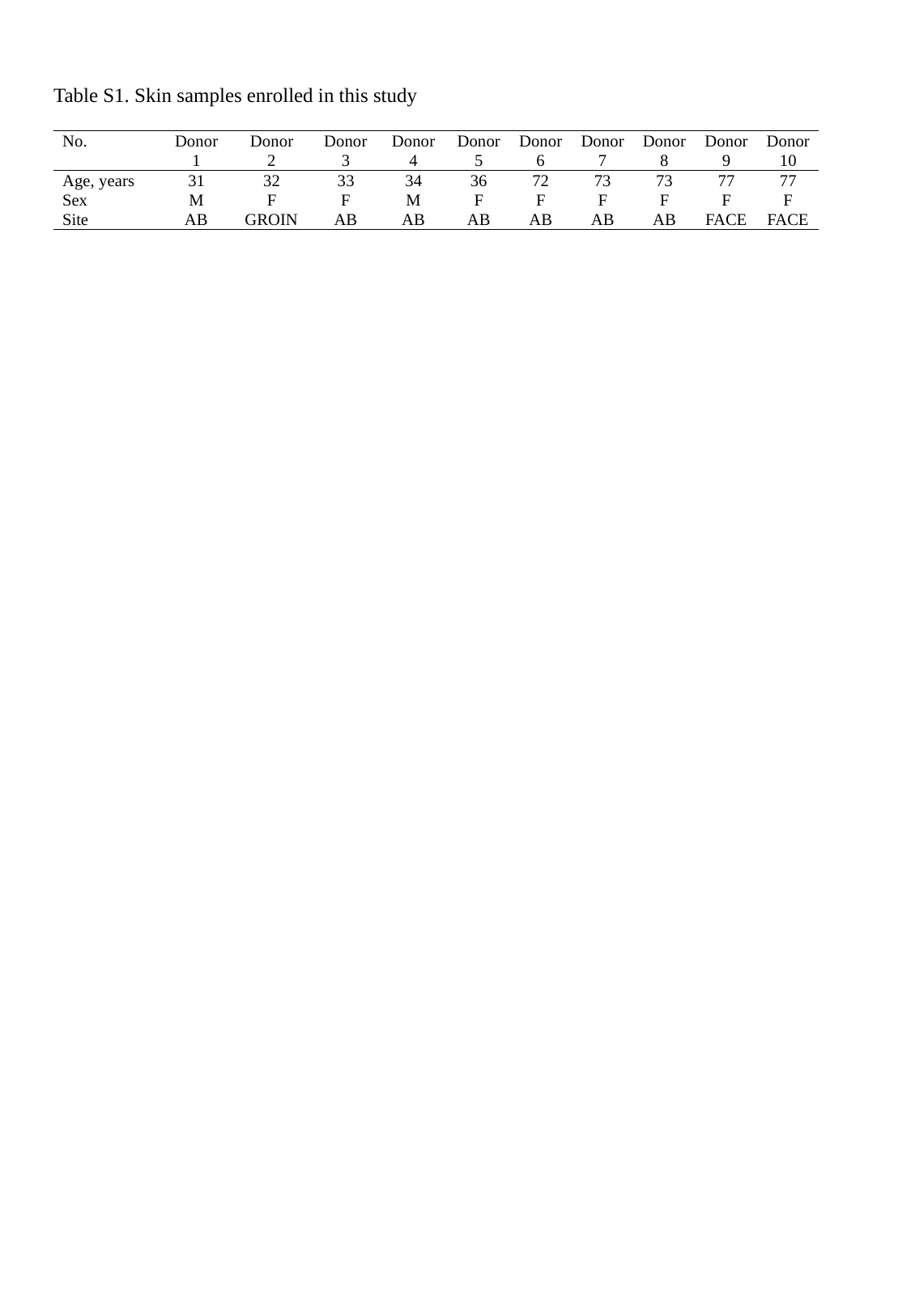

Supplement: Supplementary file 1 — FIGURE S1: (A) Venn view of DEGs from FB (blue), KTd (yellow) and Ktu (purple). (B) Enriched Wikipathways in FB (HNF) and KT (HNK). “Count” indicate gene number modulated in each pathway. TABLE S1: Description of the skin samples enrolled in this study. [file JOCD-25-e70708-s001.pptx]
